# Systemic review of the epidemiology of autism in Arab Gulf countries

**Published:** 2014-10

**Authors:** Huda O. Salhia, Lubna A. Al-Nasser, Lama S. Taher, Ali M. Al-Khathaami, Ashraf A. El-Metwally

**Affiliations:** *From the Department of Epidemiology and Biostatistics (Salhia, Al-Nasser, Taher, Al-Khathaami, El-Metwally), College of Public Health and Health Informatics, King Saud bin Abdulaziz University for Health Sciences, the Alghadeer Primary Healthcare Center (Salhia), General Directorate for Primary Healthcare Centers, Ministry of Health, the Dental Services, Central Region (Al-Nasser), the Neurology Department (Al-Khathaami), King Abdulaziz Medical City, Riyadh, Kingdom of Saudi Arabia, and the Epidemiology Group (El-Metwally), The Institute of Applied Health Sciences, University of Aberdeen, Aberdeen, Scotland, United Kingdom*

## Abstract

**Objective:**

To assess the current state of knowledge on the epidemiology of autism in Arab Gulf countries, and identify gaps for future research.

**Methods:**

PubMed and ScienceDirect databases were used to identify relevant articles published until the 3rd of April 2013 (date of search). The search was conducted using the electronic library of King Saud Bin Abdulaziz University for Health Sciences, Riyadh, Saudi Arabia. Studies were eligible for inclusion if they concerned the epidemiology of autism, conducted in any Arab Gulf country, and published in English.

**Results:**

Twelve articles met the inclusion criteria. Studies showed a prevalence ranging from 1.4 to 29 per 10,000 persons. Identified risk factors were metabolic, autoimmune, and environmental in nature. The following determinants were found as possible contributing factors for autism: suboptimal breast-feeding, advanced maternal and paternal age, cesarean section, and prenatal complications.

**Conclusion:**

Only a few studies explored the epidemiology of autism in Arab Gulf countries and none have investigated the burden of the disease on the child, family, or society. More research is needed to better identify the burden and risk factors of autism in Gulf countries.

Autism or autistic disorder is a neurodevelopmental disorder that impairs a person’s ability to communicate, interact socially with others, and respond to certain stimuli in their surroundings. The condition is usually diagnosed by the age of 3 and is more prevalent in males than females.^1^ Other closely related terminologies are autism spectrum disorders (ASD), and pervasive developmental disorders (PDD). Autism spectrum disorder is characterized by delayed language development, repetitive and stereotyped patterns of behavior, imagination, and hindered social interaction,^2^ while PDD refers to a group of conditions that include autistic disorder, Rett’s disorder, Asperger’s disorder, as well as a group of other related conditions.^3^ The first studies on autism were circulated in the 1960s, and many less severe types of autism were not identified until the 1980s. Since then, epidemiologists have conducted numerous surveys on autism that yielded higher prevalence rates year after year.^4^,^5^ This might be attributed to: increased awareness by both healthcare professionals and families of autistic children, and changes in diagnostic criteria. However, it is likely that the prevalence remains underestimated as many cases of autism are probably undiagnosed or unrecognized in the community, particularly the mild ones. This might be partly due to lack of awareness of both public and health care providers. Lack of screening programs and difficult access to care due to various reasons might have also contributed.^6^ Although there is little research into the global burden of autism, some studies in the USA and UK estimated the annual cost of autism on the economy and community to be more than several billion US dollars.^7^,^8^ The prevalence of autism is variable; Europe reported a median of 18.75 per 10,000, and the USA reported a median of 21.6 per 10,000. However, China reported a lower median of 11.6 per 10,000. Similarly, the male to female case ratio ranged from 1.33:1 to 16:1.1 Socioeconomical factors affect the prevalence rate prominently. For instance, studies in India have shown that most diagnosed cases belong to middle-class families. Upper class families do not frequent public health centers to treat autistic children, and families from low socioeconomic strata do not access such facilities unless the child is acutely ill.^9^-^11^ Obviously, the global prevalence for PDD was higher than that of autistic disorder due to the more inclusive definition of PDD.

A multitude of heritable and non-heritable exposures were studied in relation to ASD.^12^ Based on extensive review of ASD epidemiology; Newschaffer et al^12^ suggested a model that categorized potential risk factors into: 1) genetic predisposition of the mother, 2) environmental factors acting the mother, 3) genetic predisposition of the child, and 4) environmental factors affecting the child. Early diagnosis and subsequent intervention for ASD and PDD are paramount, as research has shown the potential of greater benefit with early intervention.^13^-^15^

The Gulf Cooperation Council (GCC) countries comprise 6 Arab countries, which are located in the Arab peninsula; namely: Bahrain, Kuwait, Qatar, Saudi Arabia, Sultanate of Oman, and United Arab Emirates (UAE). These countries have a shared geographical location, ethnic backgrounds, and life styles. Moreover, genetic exposures such as consanguinity and multiparity is common in this area. Epidemiological research into autism in the GCC is relatively new, and the burden of autism in this part of the world is still unclear. Our objective was to review the current state of knowledge of epidemiology on autism in the GCC, and make recommendations for future research.

## Methods

In this review, PubMed and ScienceDirect databases were used to identify relevant articles published until the 3rd of April 2013 (date of search). The search was conducted using the electronic library of King Saud Bin Abdul Aziz University for Health Sciences, Riyadh, Saudi Arabia. PubMed search identified 3 groups of articles as follows: disease of interest (keyword: autism), geographical location (keywords: Saudi, UAE, Oman, Kuwait, Qatar, and Bahrain), and epidemiological terms (keywords: epidemiology, prognosis, diagnosis, pattern, odds, risk, incidence, prevalence, impact, trends, and biomarker). Finally, the Boolean operator AND combined the results for each group of articles, a total of 22 articles met the inclusion criteria. For the ScienceDirect database, a search was conducted for the key words “epidemiology of autism” and each of the GCC Countries individually, which yielded a total of 79 results.

**Figure 1:**
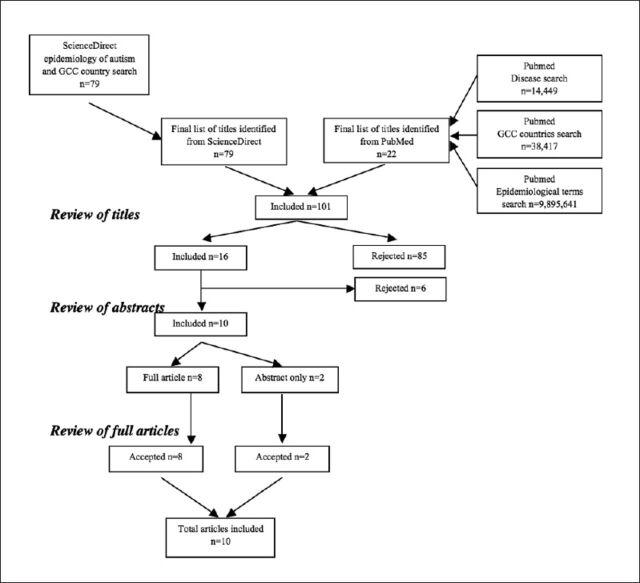
Literature search flowchart.

The titles of the final list consisting of 101 results were reviewed, 85 articles were excluded because they were not related to epidemiology of autism, and the abstracts of the remaining 16 articles were obtained. After reviewing the 16 abstracts, 6 more articles were eliminated: reviews (n=3), 2 were qualitative studies and abstract/full text of the sixth was irretrievable. A search was conducted for the full text of the remaining 10 articles, of which 8 were located and 2 were not. Thus, 8 full articles and 2 abstracts were used in this review. Two authors carried out data extraction and results synthesis independently. The complete search strategy is illustrated in **Figure 1**. Two more articles, both based on the same study, were included. The first was an article published as a brief communication,^16^ and the second is an article published during the writing up of this review.^17^

## Results

Studies were identified from 3 GCC countries only; Oman, Saudi Arabia, and UAE. No articles were identified from Bahrain, Kuwait, or Qatar. From the 10 included articles, 3 were prevalence studies, and the rest discussed potential risk factors or biomarkers for autism in patients from GCC countries.

### Prevalence studies

Prevalence studies were conducted in UAE,^18^ Saudi Arabia,^19^ Oman,^20^ and Bahrain.^17^ The prevalence of ASD was 1.4 per 10,000 in Oman, and 29 per 10,000 for PDD in UAE, and 4.3 per 10,000 in Bahrain.^17^,^18^,^20^ The Saudi study documented patients’ characteristics and reasons for referral for group of Saudi autistic patients.^19^ Male gender and history of developmental delay were significantly associated with autism prevalence in all 3 studies. Consanguinity was present in 28.6% of Saudi patients, and behavioral problems such as hyperactivity or aggression were evident in 45% of patients (**Table 1**).^19^

**Table 1:** Summary of epidemiological studies on autism/autism spectrum disorder in the Gulf Cooperation countries.

Author, Year, Ref	Country	Study design	Population	Sample size	Diagnostic criteria	Main findings
***Prevalence studies***						
Eapen et al, 2007^18^	UAE	Cross sectional	Preschool children in UAE	694 Emirati children (aged 3 years)	DSM-IV	Autism-screening questionnaire gave a prevalence of 58 per 10,000, after clinical evaluation the prevalence dropped to 29 per 10,000. Presence of autistic features was associated with males, behavioral problems, and family history of developmental delay
Al-Salehi et al, 2009^19^	Saudi Arabia	Cross sectional	Children in Saudi Arabia	49 children diagnosed with ASD	DSM-IV CARS	Communication problems present in 71% of patients, consanguineous marriages 28.6%, male:female ratio 3:1, mean age at referral 6.3 years, behavioral problems present in 45% of patients. Most patients were self-referred or from primary care givers
Al-Farsi et al, 2011^20^	Oman	Cross sectional	All children in Oman aged 0-14 years	800,000	DSM-IV CARS	Prevalence 1.4 per 10,000 (95% CI: 1.2-1.7), prevalence was 2.5 times more in males, age-specific prevalence was highest among children aged 5-9 years
Al Ansary & Ahmed, 2013^17^	Bahrain	Case-control (case identification phase)	All children in Bahrain	Records from the only referral clinic in Bahrain from 2000-2010 (N = 100 cases)	DSM-IV-TR	Prevalence 4.3 per 10,000 population, with a male:female ratio of 4:1.
***Risk factors/Biomarkers studies***						
Al-Gadani et al, 2009^24^	Saudi Arabia	Case-control	Children with autism in Saudi Arabia (age 3-15 years)	30 cases, 30 (age and gender matched) healthy controls	DSM-IV	Lipid peroxidation, GSH-Px, and SOD were significantly higher in autistic patients while vitamin E and glutathione levels were lower. Vitamin C and catalase showed insignificant differences between groups.
Al-Mosalem et al, 2009^25^	Saudi Arabia	Case-control	Children with autism in Saudi Arabia (age 3-15 years)	30 cases, 30 (age and gender matched) healthy controls	DSM-IV	Significant increases in activity of Na(+)/K(+)ATPase (148.8%) and lactate levels (40%) among autistic patients
Ali et al, 2011^27^	Oman	Case-control	Children with autism in Oman (age 3-5 years)	40 ASD diagnosed cases, 40 controls (age and gender matched)	DSM-IV CARS	Serum homocysteine level among ASD patients was significantly higher than controls and reference range. Serum folate and Vitamin B^12^ levels were lower in autistic patients than controls
El-Ansary et al, 2011^26^	Saudi Arabia	Case-control	Children with autism in Saudi Arabia (age 4-12 years)	26 ASD cases, and 26 age-matched controls	ADI-R ADOS 3DI	Fatty acid profile in autism patients was altered compared with controls. Most saturated fatty acids showed markedly increased levels while levels of polyunsaturated acids were lower in autistic patients. Area under the curve for the receiver-operating curve for specific fatty acids ranged from 0.611 to 1.0
El-Ansary et al, 2011^22^	Saudi Arabia	Case-control	Children with autism in Saudi Arabia (age 4-12 years)	25 autism cases, and 16 healthy controls (age-matched)	ADI-R ADOS 3DI	Autistic patients showed significantly higher blood lead level (Pb^+2^). Plasma levels of neurotransmitters (GABA, 5HT and DA) were elevated
Al-Ayadhi & Mostafa, 2011^23^	Saudi Arabia	Case-control	Children with autism in Saudi Arabia	42 autistic children and 42 healthy controls	CARS	Significantly elevated serum osteopontin found in 81% of autistic patients. Severe autism showed higher osteopontin than mild or moderate autism. Osteopontin levels were positively correlated to CARS scores.
Al-Farsi et al, 2012^21^	Oman	Case-control	Children with ASD in Oman (aged 3-14 years)	102 ASD diagnosed children, and 102 healthy controls	DSM-IV CARS	Increased risk of ASD was found in relation to: late initiation of breastfeeding (OR=1.47, 95% CI: 1.01-3.1) and no colostrum intake (OR=1.7, 95% CI: 1.03-4.3). Exclusive breastfeeding and continued breastfeeding up to 24 months significantly decreased risk of ASD
Al Ansary & Ahmed, 2013^17^ and Al Ansary & Ahmed, 2012^16^	Bahrain	Case-control	Children with ASD in Bahrain	350 Age and gender-matched controls nocturnal enuresis, mild behavior disorder and no psychopathology	DSM-IV-TR	Significant association with advanced maternal age above 30 years (OR = 1.83, CI: 1.02-3.28) and paternal age above 30 years (OR = 2.08, 95% CI: 1.15-3.7). Non-significant relation with birth order. Significant relationship with delivery by cesarean section and having mothers who suffered from prenatal complications

DSM-IV - Diagnostic and Statistical Manual of Mental Disorders, 4th edition, DSM-IV CARS - Childhood Autism Rating Scale, DSM-IV-TR - Diagnostic and Statistical Manual of Mental Disorders, 4th edition, text revision, ADI-R - Autism Diagnostic Interview-Revised, ADOS - Autism Diagnostic Observation Schedule, 3DI - Developmental Dimensional Diagnostic Interview, GSH-Px - glutathione peroxidase, SOD - superoxide dismutase, Na(+)/K(+)ATPase - sodium-potassium adenosine triphosphatase, GABA - gamma-aminobutyric acid, 5HT - 5-hydroxytryptamin, DA - dopamine, OR - odds ratio, CI - confidence interval

### Risk factors/biomarker studies

The included studies were conducted in Saudi Arabia, Oman, or Bahrain, and all employed the case-control study design. Sample size varied between these studies from 52 to 204 patients; and the total number of autistic patients reviewed was 395. The risk factors investigated were: suboptimal breastfeeding,^21^ lead exposure,^22^ serum osteopontin,^23^ maternal and paternal age, cesarian section, and prenatal complications. Delayed initiation of breastfeeding and no colostrum intake was associated with increased risk of ASD. Exclusive and prolonged breastfeeding (up to 24 months or more) markedly reduced the risk of developing ASD.^21^ Higher blood levels of lead^22^ and osteopontin^23^ were found in autism patients. Occurrence of autistic disorder was correlated with maternal (OR 1.83) and paternal (OR 2.08) age above 30 years at time of birth. The disease was also more common among children of mothers who delivered with a cesarian section or had antenatal complications^16^,^17^ (**Table 1**).

Various biomarkers profiles were altered among autistic patients compared with their healthy controls. Lipid peroxidation,^24^ glutathione peroxidase,^25^ superoxide dismutase, sodium-potassium adenosine triphosphatase,^21^ lactate, saturated fatty acids,^26^ and homocysteine^26^ levels were significantly higher among autistic patients. Inversely, levels of vitamin E,^24^ glutathione,^24^ folate, vitamin B12,^27^ and some polyunsaturated acids were much lower in autistic patients. Serum osteopontin levels were higher among autistic patients,^25^ and levels were positively related to the severity of autism (**Table 1**).

## Discussion

The prevalence of ASD and PDD was highly variable between included studies.^18^,^20^ The difference between both rates could be attributed, in part, to setting, method of assessment, and age of participants. The Omani study^20^ included a larger sample with wide age range to determine prevalence of formally diagnosed ASD from medical records. Conversely, the Emirati study^18^ screened a sample of preschool children for undiagnosed PDD, and the case definition for PDD is more inclusive than ASD. This fluctuation in prevalence is consistent with other studies around the world. For example, studies in Europe recorded a prevalence that ranged from 1.9 up to 72.6 per 10,000, and the range has been wider (2.8-94 per 10,000) in China.^1^

Prevalence is also affected by accessibility to a specialized autism care center and source of case identification (mainly families). A low prevalence rate is expected in areas with no facilities or centers for case reporting. Over time, healthcare professionals and families have learned more about the disorder, and the increased awareness resulted in more comprehensive diagnostic criteria. Consequently, prevalence figures would be expected to increase. This emphasizes the need to establish a reliable autism-screening tool that can be applied in community-based studies to produce more precise prevalence in GCC countries. Collaborative efforts should target increasing awareness of the community toward ASD to encourage early consultation and diagnosis. Also, availability and accessibility to diagnosis and treatment centers should be assessed in light of the burden of this disorder.

The male to female ratio in GCC countries was consistent with studies from elsewhere showing male predilection,^1^ and there is no evidence to date that explains this finding.^12^ One possible reason is that female children are more able to mask their behavioral difficulties than males.^19^ Moreover, culture in developing countries may be a contributing factor, as some families may pay more attention to the development of male children compared with females. As the burden of reporting cases falls on parents; there could be a lack of detection or lack of willingness to report certain behavior exhibited by a female child. This cultural perception could explain the older age for females at autism diagnosis in Saudi Arabia.^19^ Either way; more research is needed to explain other reasons behind this significant gender difference. Consanguinity was not related significantly to autism. Still, with consanguinity rates in Saudi Arabia that range from 34-80%, depending on rural, or urban setting, this risk factor deserves further investigation.^19^,^28^

Lead toxicity has been associated with autism,^22^ which is in agreement with other research.^29^ However, the nature of environmental exposure that might have contributed to lead toxicity was not clarified in the study. A strong dose-response protective effect of breastfeeding^21^ is in agreement with other studies,^30^,^31^ but potential for recall bias remains high as data collection depends on parents’ memory and reporting. Findings for various biomarkers were comparable with studies carried out elsewhere.^29^,^32^-^35^ Still, more research is needed to clarify applicability of these biomarkers for early screening or monitoring of autistic patients.

Most reviewed studies shared 2 limitations. First, is the sample size, as numbers of cases and controls used were small and cannot be considered representative. Also, the recruitment of cases and controls was from specialized clinics/tertiary care institutions, which might be problematic when extrapolating results to community autistic children in GCC countries. Second, is that cases were recruited from pediatric wards, and none come from psychiatric clinics. The diagnosis of autism must include more comprehensive behavioral/psychological considerations to form a full picture about the disorder. The current study’s limitations were the small number of studies identified and absence of studies from 3 GCC countries, which precluded comparing prevalence of autism across countries.

### Future direction of autism research

Generally, there is an evident lack of research into risk factors affecting the etiology of autism in GCC countries. Neither genetic nor environmental exposure have been studied in detail. Consanguinity, multiparity, and closely-spaced pregnancies are common in the GCC region, and provide an exceptional opportunity to learn more about genetic determinants of the disease. Also, dietary habits should be looked into as they could help in i) investigating the risk and prognostic factors of the disease and, ii) assisting families by identifying high-risk foods that could affect their children. More focus should be given to children in psychiatric wards to identify autistic symptoms among them.

In conclusion, population-based studies should focus on quantifying the burden of ASD in GCC countries. Knowing the burden and extent of disease could help design screening tools that are applicable, culturally acceptable, and cost-effective to identify individuals who can benefit the most from early diagnosis and intervention. Furthermore, raising ASD awareness among parents, preschool/elementary school teachers are invaluable in helping autistic children cope with different challenges.
